# Allergic and intolerance reactions to wine 

**DOI:** 10.5414/ALX01420E

**Published:** 2018-09-01

**Authors:** B. Wüthrich

**Affiliations:** Specialist for Allergology and Clinical Immunology, Specialist for Dermatology, Zollikerberg, Switzerland

**Keywords:** wine, hypersensitivity reactions, allergies, intolerances, ethanol metabolism, aldehyde dehydrogenase, LTP Vit v 1, Botrytis cinerea, fining agents, insect proteins, sulfites, histamine

## Abstract

Hypersensitivity reactions to alcoholic beverages (particularly red wine) are relatively frequent, affecting 10% of the general population. Hypersensitivity reactions due to alcoholic drinks, mainly in the form of airway reactions (rhinitis and asthma), occur significantly more frequently in persons with pre-existing rhinitis and asthma. In terms of pathogenesis, it has to be differentiated between immunologic, mainly IgE-mediated, hypersensitivity reactions (wine allergies), and intolerance reactions in which no causative allergen-specific immune mechanisms can be detected. Allergens responsible for wine allergy could be: grape (*Vitis vinifera*) proteins (particularly the major allergen lipid transfer protein Vit v1), proteins and ingredients used for the fining of wines such as fish gelatin or isinglass (swim bladder of the fish huso, family of sturgeons), ovalbumin, dairy (casein) products, gum arabic, enzymes (lysozyme, pectinase, glucanase, cellulase, glucosidase, urease, aromatic enzymes), molds (particularly *Botrytis cinerea*) responsible for the noble rot in wines, yeasts and proteins from insects that contaminated the mash. Type 1 allergic reactions (positive prick tests) have been described for inorganic components like ethanol, acetaldehyde, acetic acid and sulfites, but no specific IgE could be detected in the serum. Ethanol, acetaldehyde and acetic acid, flavonoids (anthocyanins and chatechines), sulfites, histamine and other biogenic amines are the main causative agents of intolerance reactions (pseudoallergic reactions) to wine. After a short historic review of viticulture and the importance of wine in classical antiquity, we go into the chemical processes of alcoholic fermentation and the genetically inherited “flush syndrome” caused by an acetaldehyde dehydrogenase 2 polymorphism, subsequently we focus on the different etiologic factors of allergies and intolerance reactions to wine. The most frequent intolerance reactions to sulfites occur particularly after the ingestion of white wine and in asthma patients. Intolerance reactions to histamine and other biogenic amines occur mainly after ingestion of red wine and in persons with diamine oxidase (DAO) deficiency.

**German version published in Allergologie, Vol. 34, No. 8/2011, pp. 427-436**

## Introduction 

In a representative written survey of 6,000 individuals aged 18 – 69 years that was carried out in Copenhagen, Denmark, in 2006, the prevalence of hypersensitivity reactions following consumption of alcoholic beverages (mainly red wine) was approximately 8% (4,142 responders = 70%): 7.6% reported symptoms of the upper airways (sneezing fits, rhinorrhea, nasal obstruction), 3.2% reported symptoms of the lower airways (cough, dyspnea, asthma attacks), and 7.2% reported skin symptoms (erythema, pruritus, urticaria). The cumulative prevalence of all three organ manifestations was indicated to be 13.9% (95% confidence interval: 12.9 – 15.0%). 9.9% of the respondents had observed symptoms after drinking alcoholic beverages within the past 12 months [1]. Alcohol-induced hypersensitivity reactions of the airways were significantly more frequent in people with rhinitis and asthma (odds ratio 3.0 – 8.1; p-values < 0.001 for all associations). The editorial of the same journal warns against underestimating such hypersensitivity reactions [2]. Severe anaphylaxis after wine consumption [3], even 2 fatalities in asthma patients [4], as well as exercise-induced wine anaphylaxis [5] have been described. 

After a short review of the history of wine, the hypersensitivity reactions after wine consumption will be presented according to their pathophysiologic mechanisms and etiology. 

## A short history of viticulture 

The history of viticulture is closely related to the development of early cultures and can be traced back for almost 8,000 years. Ancient Persia is regarded to be the first wine growing culture. Shiraz, mistaken for the place of origin of the grape variety carrying the same name and situated near the Persian capital Persepolis, was famous for its wines and enjoyed the reputation of producing the best wine in the Middle East. In south Caucasus (today Georgia) and in the Middle East region Sumer (today southern Iraq), traces of viticulture date back to 5000 BCE. Subsequently, wine was cultivated in the entire Middle East. The Egyptian Pharaoh Scorpion 1 was buried with 700 jugs of wine. 

Approximately 1700 BCE, the Minoans cultivated the first noble wines on the island of Crete. In ancient Greece, wine was generally drunk with water; to drink undiluted wine was attributed to the Barbarians. In Greece, pure wine was only used for the ritual of libation at the beginning of a symposium. During the time of the Roman Empire, viticulture spread over great parts of Europe with the conquering of legions. The English term “wine”, the French “vin”, and the German “Wein” all derive from the Latin word “vinum”. Wine was considered the drink of the gods. In mythology, the Greek Dionysos and the Roman Bacchus were both gods of the wine. 

## Wine and health 

The Greek Father of Medicine, Hippocrates (460 – 375 BC), wrote in his Hippocratic Corpus “On Ancient Medicine”, which dates back to the 5^th^ century BC, “The first glass of wine is for health, the second glass of wine is for gaiety, the third glass of wine is for good sleep, and every further glass is a danger”. And then, “Wine is a thing marvelously suited to man, in health as in sickness, if it is administered appropriately, and in just measure in accordance with the individual constitution.” Hippocrates prescribed wine as a remedy for fever, gastrointestinal diseases, bleeding, absence of appetite, and strengthening convalescent patients. 

From ancient times until the 18^th^ century, when wine and all other alcoholic beverages started to be considered a health risk, wine was not only a drink but one of the most important cures. Even the Jewish Talmud cites Rabbi Banal with the saying, “Where there is a lack of wine, you need drugs”. But Hippocrates also warns, “Too much wine usually causes complaints”. He probably did not mean hypersensitivity reactions, but the famous “hangover” or alcohol intoxication. 

## Hypersensitivity reactions after wine consumption 

**Figure 1.**
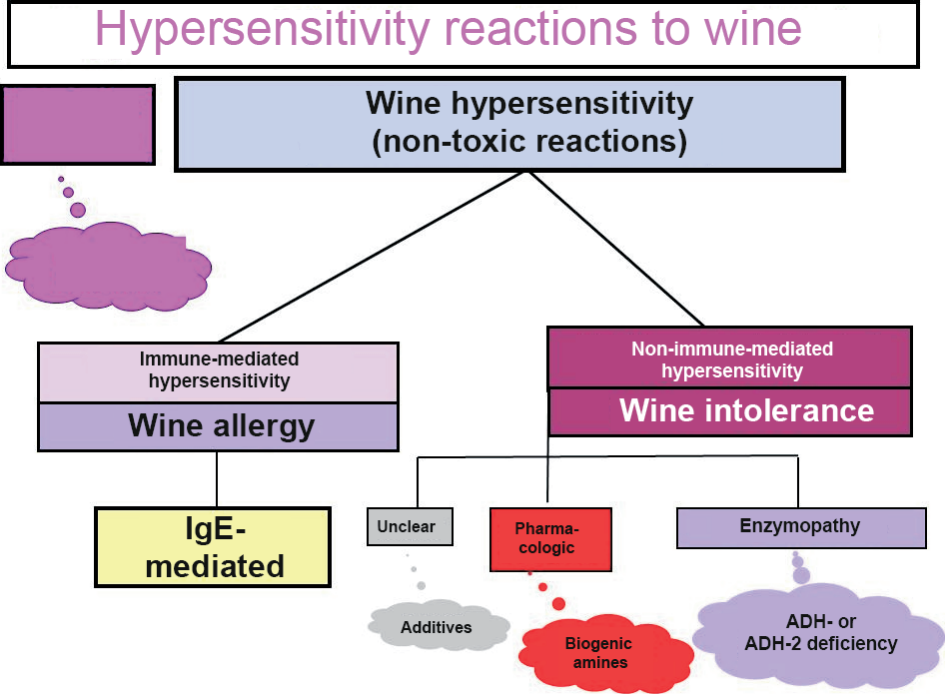
Classification of hypersensitivity reactions to wine (following the EAACI and WAO nomenclature [6, 7]).

**Figure 2.**
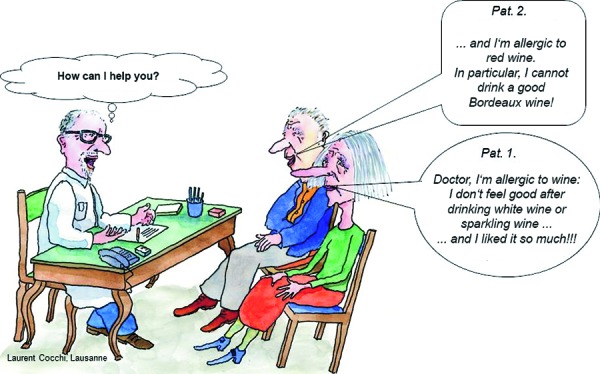
A frequent situation in daily practice: “Doctor, I’m allergic to white wine” (Patient 1) or “Doctor, I’m allergic to red wine” (Patient 2).

According to the European Academy of Allergology and Clinical Immunology (EAACI) and the World Allergy Organization (WAO), hypersensitivity is defined as follows, “Hypersensitivity causes reproducible symptoms or signs initiated by exposure to a defined stimulus at a dose tolerated by normal subjects” [6, 7]. According to this, a hypersensitivity reaction after the consumption of wine is a non-toxic reaction and needs to be differentiated from methanol intoxication. A classification is presented in Figure 1. In the context of wine, a distinction can be made between immunologically mediated wine allergy and wine intolerance. In the latter, no allergen-specific immunologic mechanisms that would trigger the reaction can be detected. Patients with hypersensitivity reactions to white or red wine always assume to be suffering from an “allergy” (Figure 2). 

## Alcohol (ethanol) metabolism and genetically determined hypersensitivity reaction 

**Figure 3.**
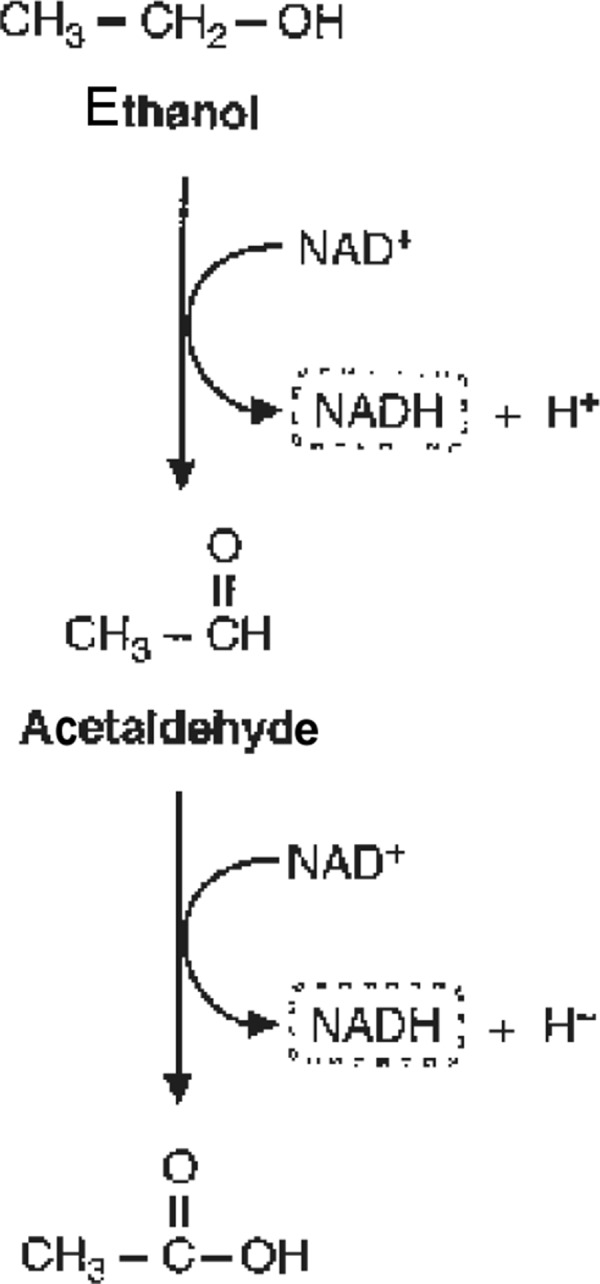
Ethanol metabolism.

In the last step of alcoholic fermentation, yeasts convert acetaldehyde (ethanal) to ethanol by the enzyme alcohol dehydrogenase (ADH). The depletion of alcohol in the liver is carried out in three steps (Figure 3):

- Ethanol (C_2_H_5_OH) is converted again to acetaldehyde (CH_3_CHO) by ADH;
- the toxic acetaldehyde is converted to acetate (C_2_H_4_O_2_) by aldehyde dehydrogenase 2 (ALDH-2);
- in the citric acid cycle, acetate is depleted to carbon dioxide and water.

Flush syndrome after wine consumption is due to an enzymopathy. First, there is a genetically determined high activity of the enzyme ADH; due to this, ethanol is very rapidly converted to high amounts of toxic acetaldehyde. Second, a genetically determined deficit of the enzyme ALDH-2 can be present so that acetaldehyde cannot be detoxified sufficiently. 46% of Japanese and 56% of Chinese are affected by acetaldehyde dehydrogenase 2 polymorphism. Mutated ALDH-2 is less effective in processing acetaldehyde than the wild-type protein and is depleted faster. Thus, toxic acetaldehyde is accumulated more easily in the body leading to symptoms of intoxication (flush syndrome) [8]. 

## Wine allergies 

Potential allergens (proteins) in wine are proteins from grape (*Vitis vinifera*), *Botrytis cinerea* (mold responsible for the noble rot in wine), other molds, yeasts, proteins and ingredients used for the fining of wine, as well as proteins from insect that have contaminated the mash. Possible Type 1 allergic reactions have also been described for non-organic ingredients like ethanol, acetaldehyde, and acetic acid, although for these haptens no specific IgE could be detected in the serum. 

### Allergies to proteins from grape (*Vitis vinifera*)

Endochitinase 4A, a lipid transfer protein (LTP), and a thaumatin have been identified as allergens in grapes as well as in wine by Pastorello et al. [9] in 3 patients without pollinosis who had experienced anaphylactic reactions to red wine and in 11 patients with grape allergy. The recently characterized LTP Vit v 1, molecular weight 9 kD, is the major allergen and cross-reacts with peach and cherry LTP. This allergen has also been identified in a German winemaker with a non-pollen-associated anaphylactic reaction to white wine [10]. The same investigators managed to induce tolerance in this patient by oral administration of increasing concentrations of white grapes. After a daily maintenance dose of 20 g grapes was reached, the patient tolerated 66.5 ml white wine in a challenge test. The 24-kd protein (thaumatin homologue) is strongly homologous to peach thaumatin and is a minor allergen. According to investigation by Pastorello et al. [9], endochitinase A4 as an allergen is present in young wines and in wine of the brand Fragolino (with strawberry flavor). 

### Molds, particularly Botrytis cinerea, as wine allergens

The mold *Botrytis cinerea* is responsible for the noble rot in grapes and is indicated to trigger “wine allergy”. However, in an extensive literature research, including the consultation of a specialist for fungus allergies [12], no documented case report could be found. Also other molds and yeasts, like *Chrysonilia sitophila, Mucor plumbeus, Penicillium glabrum, Aspergillus sp., P. olsonii und Trichoderma longibrachiatum*, which are present in cellars and on corks and can contaminate wine, are indicated as potential wine allergens in the literature [13, 14, 15]. But also for these microorganisms there are no case reports. 

### Protein-containing clearing agents as potential allergens

In winemaking, the technique of fining is used to clear and biochemically stabilize the wine. By precipitation of very small floating particles, fining prevents a later clouding of the wine. Through this, wine can resist various storage, transport, and temperature conditions and still remains stable and durable for a long time afterwards. 

The clearing of the wine is used to precipitate trub – mainly dead yeasts –, bacteria, tartrates, proteins, pectins, various tannins, and other phenolic compounds. This clearing takes place naturally (allowing to settle), but also by mechanical (separation, filtration) or physical techniques (fining). 

The following protein-containing clearing agents can be suspected as allergens and have to be declared according to Amendment IIIa of the EU Directive 2000/13/EC [16]: fish gelatin or isinglass (swim bladder of the fish huso, family of sturgeons), ovalbumin, dairy (casein) products, and gum arabic. Enzymes like lysozyme, pectinase, glucanase, cellulase, glucosidase, urease, and aromatic enzymes are not allowed in biologic winemaking. Lysozyme could be particularly problematic for patients with egg allergy [17]. Nevertheless, in a study applying double-blind, placebo-controlled oral provocation testing (DBPCOP) with wines that were cleared with egg white, isinglass, or tannins (not from grapes), no symptoms could be triggered in patients with allergies to fish, hen’s egg, or peanut [18]. As the study included too small a number of patients with milk allergy, the results could not be statistically validated and no conclusions on the allergenicity of wines cleared with milk proteins (casein) for patients with milk allergy are possible. In a second study, 5 German wines were cleared with high doses of egg white, lysozyme, milk casein and fish gelatin, or isinglass. Subsequently, parallel samples were filtered [19]. 14 patients with allergy to hen’s egg (n = 5), cow’s milk (n = 5), or fish (n = 4) were prick-tested with the clearing agents as well as with samples of filtered, cleared, and non-cleared wine. Finally, a DBPCOP with wines was carried out in all patients. The skin prick tests were positive for hen’s egg (n = 5), ovalbumin (n = 5), lysozyme (n = 4), cow’s milk (n = 5), casein (n = 4), and codfish (n = 3), but not for fish gelatin (n = 0). Positive skin prick tests were detected for wines cleared with ovalbumin (n = 3), lysozyme (n = 2), casein (n = 1), gelatin (n = 0), and isinglass (n = 3) as well as for uncleared wines (n = 1 – 2 in each patient group) without statistically significant differences between the groups. Oral provocation tests were negative for all wine samples. Thus, the authors conclude that although wines with high concentrations of clearing agents resulted in positive skin prick tests in patients with the corresponding allergy, wines in which clearing agents were used do not cause problems in allergic persons when filtered. 

### Proteins from insects (hymenoptera) as wine allergens

In a spectacular report by Spanish authors [20], 5 patients developed allergy symptoms after drinking young wine (3 × OAS and face flushing, 1 × asthma, 1 × anaphylactic shock). Skin prick testing with the suspected wine was positive, but testing with samples of an older wine was negative [20]. Oral provocation testing with the young wine was positive (OAS and flush; FEV_1_-reduction by 25%); oral provocation tests with other types of wine were negative. The patients had positive IgE-antibodies against venom of *Vespula* or *Polistes* wasps as well as against the suspected wine. There was no history of insect stings. In immunoblot insect venom allergens could be detected. Inhibition testing with *Polistes* extract was positive. When grapes are pressed, insects can contaminate the mash. Their venoms are decomposed during the fermentation of old wines. The authors postulate that the sensitization that was necessary to induce the anaphylactic reaction took place orally. 

### Cross-reactive carbohydrate determinants (CCD)

Carbohydrate determinants are frequent in plants and invertebrates. They are composed of 90 – 95% glycoproteins, i.e., proteins with carbohydrate (sugar). Glycoproteins contain one or more complex oligosaccharide chains that bind to the peptide structure of the proteins. As glycoproteins can have a significant structural homology beyond the limits of protein families, these structures are responsible for extensive cross-reactivity. The term “cross-reactive carbohydrate determinants” or CCD derives from this characteristic. It is currently being discussed whether or not IgE-antibodies against carbohydrate epitopes of glycoproteins play a clinical role. Spanish authors have investigated the prevalence of specific serum IgEs to CCD (MUXF3, the N-glycan of bromelain – bromelain is a glycoprotein extracted from pineapples and is used to clarify cross-reactivities between glycans and other glycoproteins), pollen (*Lolium perenne* and *Olea europaea*), hymenoptera venoms (*Apis mellifera* and* Vespula spp.*), and house dust mite (*Dermatophagoides pteronyssinus*) in a sample of 457 adults (218 alcohol abstainers, 195 mild-to-moderate drinkers, 44 heavy drinkers) and in a further 138 alcoholics [21]. 5.6% of the sample population (95% CI: 3.5 – 7.6%) had positive IgE values > 0.35 kU/l for CCD. These sCCD-IgE titers were particularly high in heavy drinkers who also showed a high prevalence of positive IgE results against pollen and insect venom; this IgE sensitization was closely correlated with the presence of Anti-CCD-IgE. Inhibition tests confirmed these cross-reactivities. The authors conclude that in drinkers the impact of sIgE results for CCD has to be interpreted with caution. Commins and Platt-Mills [22, 23] have detected IgE-antibodies specific for oligosaccharides of mammal (galactose alpha-1, 3-galactose) in 2 patients with anaphylactic reactions after meat ingestion. Thus, in so-called idiopathic anaphylaxis, specific anti-CCD-IgE should be determined [22, 23]. 

### Allergic reactions to inorganic wine components (ethanol, acetaldehyde, acetic acid, and sulfites)

In very few cases of hypersensitivity reactions after wine drinking, skin prick testing showed positive immediate-type reactions to ethanol or its metabolites – via acetaldehyde dehydrogenase – acetaldehyde and acetic acid as well as to sulfites [24, 25, 26, 27, 28]. There were also scattered cases with positive histamine release tests. However, specific IgE-antibodies against these haptens could not be detected so far. Intolerance reactions (pseudoallergies) are more frequent (see below) [29, 30, 31, 32]. 

## Intolerance reactions to wine 

Ethanol, acetaldehyde and acetic acid, flavonoids (anthocyanidins and catechins), sulfites, histamine and other biogenic amines are the main triggers of intolerance reactions to wine (pseudoallergic reactions). 

### Intolerance reactions to ethanol, acetaldehyde, and acetic acid

With the exception of genetic flush syndrome in reaction to ethanol (see above), these anaphylactoid reactions – frequently in the form of urticaria – are non-allergic hypersensitivity reactions [29, 30, 31, 32]. Skin prick tests are negative; diagnosis can only be made by oral provocation tests, preferably using the DBPCFC method. 

### Fusel alcohol

These are long-chain alcohols and other compounds that are particularly frequent in extract-rich wines. They are depleted slowly and have anesthetic effects. They are responsible for the hangover. Wine usually contains only low amounts of fusel alcohols, they can however become a problem in cases of bad fermentation. 

### Tannnin and flavonoids

Tannin consists of polymerized flavonoid phenols like catechin, epicatechin, anthocyanin, etc. Correspondingly, they are polymeres with monomeric units consisting of phenolic flavans, mostly catechin (flavan-3-ol). Red wine contains phenolic flavonoids and anthocyanidins and catechins belong to this group. They are responsible for the color of red wine. These flavonoids inhibit the enzyme catechol-O-methyltransferase and prolong the catecholamine activity. In addition, the enzyme phenol sulfotransferase (PST) is inhibited. As a result, the body can no longer detoxicate certain phenols that pass from the bloodstream into the brain where they cause migraine. Patients who suspect red wine to cause migraine attacks have indeed very low PST enzyme activity in the blood [33, 34]. Red wine tops the list of suspected food [35, 36]. In a blinded study in 19 patients who had indicated being sensitive to red wine, it was shown that it is not the alcohol content but rather components of the red wine that lead to migraine. The subjects received either 0.3 l of red wine or a vodka lemonade mix with the same alcohol content. The taste was masked by cooling and having the subjects drink with a drinking straw from a brown glass. 9 of the 11 red wine drinkers reacted with an immediate migraine attack, but none of the vodka drinkers. 5 healthy control subjects tolerated the red wine without side effects [33, 34]. The English investigators suspect polyphenols to be responsible for the migraine attacks. Red wine can contain more than 1 g/l (mainly flavonoids like catechins and anthocyanins), while white wine usually does not contain more than 250 mg/l. This theory is corroborated by the observation that, in addition to red wine, mainly chocolate is indicated to trigger migraine attacks. Approximately 12 – 18% of cocoa dry mass consist of polyphenols. Tannins, catechins, and anthocyanins also play an important role. Other authors, on the other hand, suspect tyramine (review in [36]) and histamine [37, 38] to be responsible for migraine attacks. 

### Sulfites

Sulfurization (SO_2_) of wine – which was already carried out by the old Romans – prevents the wine from turning brown and the development of harmful microorganisms like vinegar bacteria, wild yeasts, and molds. Sulfites (EC-No. 220 – 227) in wine must be declared since January 1, 2008 if their concentration is higher than 10 mg/l SO_2_ (declarations: “contains sulfites” or “contains sulfur dioxide”). The maximum allowed values in the EU are 160 mg/l for red wine and 210 mg/l for medium sweet white wine. 

Particularly in white wine, allergy-like intolerance reactions are caused by sulfite [39, 40]. Asthma patients, mostly those with the non-IgE-associated type and unstable badly controlled asthma, are especially sensitive. The so-called irritant receptors in the airways are stimulated by the sulfur dioxide generated in the stomach, which results in bronchoconstriction. Patient 1 in Figure 2 is a typical example for this. Real sulfite allergies are also possible but rather rare [28, 41] (see above). 

### Histamine and biogenic amines

**Figure 4.**
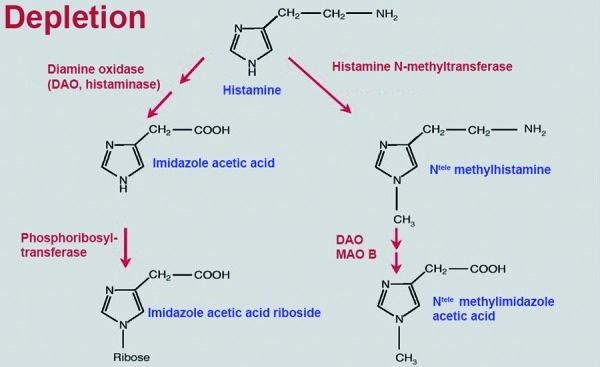
Histamin metabolism.

Biogenic amines like histamine, tyramine, cadaverine, putrescine, spermin, and spermidine are formed during the production of wine, champagne, and fruit juice by malolactic fermentation, also called biologic acid depletion. Malolactic fermentation is a secondary fermentation and follows the primary, alcohol-producing fermentation. For wine production, *Oenococcus oeni* is of particular importance as are *Lactobacillus spp., Pediococcus spp.,* and yeasts. Higher histamine concentrations can be traced back to deficient hygiene in the cellar or to uncontrolled malolactic fermentation. Another factor can be grape varieties that are sensitive to mildew and that upregulate the content of biogenic amines or their degradation products (H_2_O_2_ and aldehydes) to protect themselves against plant pathogens. Histamine can be removed by bentonite, but never completely. Furthermore, histamine content varies widely among wine types. The lowest amount of histamine is contained in rosé and white wine; champaign can have higher amounts of histamine [42]. The human body is usually able to tolerate higher amounts of externally supplied histamine and other biogenic amines. Histamine is depleted by the enzyme diamine oxidase (DAO) in the gastrointestinal tract (Figure 4). DAO is mainly present in the small intestine (terminal ileum), in the liver, kidneys, and in mast cells. DAO is continuously produced and released into the intestines. Thus, in healthy individuals a high amount of histamine can be depleted in the intestines, and DAO does not only metabolize histamine but also other biogenic amines (higher affinity). A whole series of symptoms (sneezing fits, gastrointestinal disorders, urticaria, and particularly headache – sometimes even migraine-like) can be observed in histamine intolerance syndrome [42]. The elderly Patient 2 in Figure 2 suffers from histamine intolerance. Alcohol inhibits DAO-activity and thus the depletion of histamine and other biogenic amines and increases the permeability of the intestinal walls so that histamine and other biogenic amines, that are ingested with food or the alcoholic beverage can be entrained in the blood and cross the blood-brain barrier. Histamine binds to H_3_-receptors of the small brain vessels which results in vasodilation and histamine-related headache. For this reason, the combination of alcohol and histamine-rich food (alcohol and cheese) is particularly badly tolerated by patients with histamine intolerance. A typical situation where acute symptoms can occur is the so-called “buffet situation” where people consume plenty of food and beverages that contain high amounts of biogenic amines. Chronic symptoms will frequently result from a hereditary or acquired DAO-deficit. 

## Summary and conclusion 

With an estimated prevalence of approximately 10%, intolerance reactions after drinking wine (wine hypersensitivity) are relatively frequent. The underlying pathomechanisms and etiologic factors are manifold. After exclusion of enzymopathies (acetaldehyde dehydrogenase-2 deficiency), allergic, IgE-mediated reactions as well as non-immunologic intolerance reactions occur. Many components of wine (proteins from grapes, yeasts, molds, clearing agents, ethanol, acetaldehyde, flavonoids, sulfites, biogenic amines, etc.) can trigger these reactions. The most frequent reactions are intolerance reactions to sulfites, which occur particularly after the ingestion of white wine and in asthma patients, and to histamine and other biogenic amines, mainly after ingestion of red wine. In order to be able to recommend suitable prophylactic measures, the physician should carry out a thorough diagnostic work-up in the patient. A strict pharmacotherapy of asthma or rhinitis is necessary and emergency medication should be provided to the patient. For special social events, sparkling wine with low histamine and sulfite content (e.g., Schlumberger [43]) can be recommended to the patient (Figure 2); in cases of histamine intolerance, an additional DAO substitution (Daosin®) can be applied [44, 45].
